# Challenges for palliative care day services: a focus group study

**DOI:** 10.1186/s12904-020-00699-7

**Published:** 2021-01-12

**Authors:** Felicity Hasson, Joanne Jordan, Laurie McKibben, Lisa Graham-Wisener, Anne Finucane, Kathy Armour, Shazia Zafar, Alistair Hewison, Kevin Brazil, W. George Kernohan

**Affiliations:** 1 Ulster University, Institute of Nursing and Health Research, Shore Road, Newtownabbey, BT37 0QB Northern Ireland; 2 School of Psychology, Queen’s University Belfast, Belfast, BT7 1NN Northern Ireland; 3 Marie Curie Hospice, Frogston Road West, Edinburgh, EH10 7DR Scotland; 4 Marie Curie Hospice, Marsh Lane Solihull, West Midlands B91 2PQ England; 5 University of Birmingham, Institute of Clinical Sciences, School of Nursing, College of Medical & Dental Services, Birmingham, BT15 2TT England; 6 School of Nursing and Midwifery Centre for Evidence and Social Innovation, Queens University, Belfast, BT7 1NN Northern Ireland

**Keywords:** Palliative care, Hospice program, Day care, Qualitative research

## Abstract

**Background:**

Palliative care day services provide a safe environment for people with palliative care needs, enabling them to access a range of services while acting as a respite services for family caregivers. Viewed as marginal services, they are often under resourced and under researched. The aim of this study was to understand how palliative day care services contribute to client care from the perspective of management and hospice multidisciplinary teams.

**Methods:**

A descriptive qualitative study, using six focus groups conducted with staff at three United Kingdom hospices in England, Scotland and Northern Ireland. Thirty-five participants were recruited, including management and staff. Discussions were transcribed and analysed thematically.

**Results:**

Four key themes emerged: (1) variations of care, beyond heterogeneity of patients; (2) unclear referrals and inconsistent patient population; (3) recognising strengths and challenges and (4) an uncertain future. A major focus of group discussions was the model of care and the benefits of the service, however the importance of demonstrating services’ effectiveness and value for money was highlighted.

**Conclusions:**

Management and hospice staff believed day-services to be a helpful introduction to palliative care, providing both social and medical support. Economic pressures and patient demand were influencing them to move from a social model to a hybrid model. Further research is needed to understand the effectiveness of the service.

## Background

Palliative care is a holistic patient-centred approach to care at the advanced stage of disease, providing relief of physical, psychosocial and spiritual suffering and care for patients’ families [1, 2]. As the population is ageing and with increasing incidence of chronic illnesses where a cure is impossible, and medical advances, palliative care need is set to rise [3–5]. Estimates suggest 75% of those approaching end of life could benefit from palliative care [6] which translates to approximately 40 million people worldwide [7]. Hence, this has led to the development of a range of primary palliative care services such as one community model, known as palliative care day services (hereafter ‘day-services’).

Whilst no global definition for day services exists, they typically contribute to palliative care through the delivery of a range of physical, psychosocial and spiritual services to enhance quality of life [8–10]. Whilst the structure may differ across countries [11, 12], the care model may be social, medical or a hybrid based [13–18]. Delivered during the day in fixed locations, day services provide various services and activities, with recipients later returning to their own home (or to another place of care: a nursing or residential care home).

In the United Kingdom, the first day-services unit opened in 1975 [19]. During 2014–2015, approximately 190 such services were operating across England, Wales and Northern Ireland [20]. Although considered a well-established component of palliative care provision in the United Kingdom [21], the model is not based on national evidence standards and operates with no specific care delivery guidelines [22]. This has resulted in the evolution of a variable and heterogeneous service [8] which is generally nurse-led, operated and located within or adjacent to inpatient hospices, involving a varying staff mix [15]. Previous research indicates that the majority of those who access such care are, over 60 years of age, white, middle-class and already receiving palliative care [15, 17, 19, 23, 24]. Reported benefits include social interaction, physical and psychosocial support, symptom control and assessment, access to the multidisciplinary team and relief of the burden of caring on family caregivers [13, 18, 25–31]. Day-service has been described as a transformative bridge to inpatient care, providing a connection, capable of allaying fears about inpatient palliative care and enabling earlier access to resources for care and support [32, 33].

The evidence base supporting day-services is dated and limited [28, 31, 32]. Two systematic reviews synthesising research on day-services have highlighted several problems, including poor study design and a scarcity of quantitative studies [34, 35]. There is a growing body of recent empirical data evaluating models of care reporting specific quality indicators [36] and international models from, Belgium [25] and Norway [26]. Vandaele et al. [25] report that senior Belgian day-services staff claim they offered a unique customised model of care, yet service delivery was hampered by a lack of funding and poor visibility resulting in low referral rates. However, there remains little information on where day-services stand in the palliative care landscape [25] and views from those involved in its delivery are often underreported. In response, we sought to understand how palliative day care services contribute to client care from the perspective of management and hospice multidisciplinary teams engaged in service provision.

## Methods

### Design

Qualitative exploratory focus group methodology was adopted to gain a wide range of views and to stimulate reflection on and discussion about day-services [37, 38]. The Consolidated Criteria for Reporting Qualitative Research [39] guidelines were used to ensure transparency and improve rigour.

### Participants

Three day-services were included; one each from England, Scotland and Northern Ireland. Each provided care within purpose-built premises adjacent to (and sharing some clinical and non-clinical facilities with) inpatient hospice facilities provided by Marie Curie, a national charity. Inclusion criteria were hospice staff involved in the management and delivery of day-services purposively selected and recruited via senior management in each hospice. All participants who met the criteria were given an information sheet outlining the study’s goals and a consent form. No participants dropped out of the study. All focus groups included a mixture of disciplines (3–10 participants, Table 1).

**Table 1:** Focus group participants

Site	Focus group number	Number of participants	Designations
1	1	3	Lead Nurse, Day-Services Manager & Hospice Manager
	2	7	Healthcare assistant, Staff Nurse (*n*=2), Consultant, Physiotherapist, Occupational Therapist & Volunteer Coordinator
2	3	10	Principle Social Worker, Community Services Lead Nurse, Community Services Manager, Hospice Manager, Clinical Specialist Services Manager, Palliative Care Consultant, Administrative Advisor, Allied Health Professional Manager, Manager, Lead Nurse, IPU & Day-Services Manager
	4	9	Healthcare Assistant, Staff nurse, Physiotherapist, Volunteer, Community Nurse Specialist, Social Worker, Occupational Therapist, Volunteer Complementary Therapist & Community Nurse Specialist
3	5	3	Staff nurse (n=2) and Occupational therapist
	6	3	Day-Services Sister, Hospice Manager, Palliative Care Consultant

### Data collection

In total six focus groups, two at each site, were undertaken to the point to which no new themes emerged [40]. Focus groups were conducted in May and June 2016, outside of service delivery times, but during working hours, to facilitate attendance. They were undertaken by an experienced female post-doctoral researcher (JJ) and lasted between 60 and 90 min. The researcher was not known to participants prior to the data collection. Data collection occurred within each clinical site with only consenting participants and the researcher present. A semi-structured interview schedule (Table 2), was developed, informed by the literature and overarching research aims, and refined through discussion by authors.

**Table 2:** Focus group schedule

1. What is your role in or relationship with the local palliative care day services? 2. What are the main factors that facilitate or help you in your work in or engagement with palliative care day services.? (Can you give me an example of how any one of these factors affects your work?) Specific prompts for: Clinical staff re: providing outpatient care/ Management staff re: management/delivery of day-services and General Practitioners re: referring/access to day-services) 3. What are the main factors that impede or hinder you in your work in, or engagement with, palliative care day services? Can you give me an example of how any one of these factors affects your work? (Specific prompts for see above) 4a. What do you think are the main strengths & achievements of your local palliative care day services? (What factors have enabled / underpin these strengths & achievements? /How can these strengths & achievements be sustained / built on over time? What are the main threats to these strengths and achievements?) 5a. What do you think are the main weaknesses and limitations of local palliative care day services? (What factors have promoted / underpin these weaknesses and limitations? / What needs to happen for these limitations and weaknesses to be overcome? / What are the challenged in doing so?) 6. Have there been any unexpected outcomes of palliative care day services, whether good or bad? If so, what are they? 7. How do you think palliative care day services should be developed in the future, both locally and at a national level? What are the reasons for your answer? 8. Is there anything else you would like to discuss in relation to palliative care day services? (Prompt for locally, and at a national level)	

### Data analysis

Focus groups were digitally voice recorded; transcribed verbatim and anonymised, with field notes taken. Inductive data analysis process followed Clarke and Braun’s seven steps, including transcription, reading and familiarization, coding searching for themes, reviewing, defining and naming themes and finalising the analysis [41]. Two members of the research team (FH and LMcK) independently reviewed the transcripts and derived themes from the data.

### Ethical consideration

This study was sponsored by Ulster University and approved by the Health Research Authority (Ref Number: 15/NW/0944) in April 2016. Although the work was supported by Marie Curie a registered charity, funding was independent of service provision. Written informed consent was obtained from all study participants.

## Results

Thirty-five professionals associated with day-services participated (management, *n*=19; clinical, *n*=16). Participants were unable to agree on a single definition of day-services, nor what services included. Four main themes were developed: (1) variations of care, beyond heterogeneity of patients; (2) unclear referrals, inconsistent patient population and uncertain value; (3) recognising strengths and challenges and (4) an uncertain future.

### Variations of care, beyond heterogeneity of patients

Participants described each day-service and our analysis identified wide variations in structure, referral processes, accessibility, staff mix and underpinning delivery model. A range of staff operated within day-services ranging from consultants, specialist nurses, managers, allied health professionals, healthcare assistants, volunteers and therapy staff. Participants appreciated that their service relied on volunteers for transportation of attendees, delivery of meals and therapy services, as well as providing social interaction and emotional support for patients and family caregivers.

It was recognised that day-services were continually evolving, resulting in different services being offered to patients and uncertainty about what interventions to expect. There was no standard operational definition of day-services; many reflected that their origins stemmed from a social model, geared to patients’ psychosocial needs. However, a move towards maintaining and responding to the physical and functional health of the patients through medical interventions was becoming more apparent. Participants reported that nursing, medical and allied health professionals’ assessments were a necessary activity within day-services. Assessment of patients’ medical needs, prescribing drugs, ordering investigations and providing advice and signposting to other services were routine activities. For day-services to continue, a combination of the social and medical models was needed:

*“I think we’ve managed to create a social model and a clinical model by being really flexible and I think that more hospices need to look at what they’re actually doing in their day services, because a lot more day services will shut if they don’t”* (Day Hospice Manager, FG 6)

*“ … .is it a social model or is it a medical model, I would like to think that we are 50/50, but more gearing towards the specialist model of symptom management, rehab, psycho-social support, education, promotion of health, all of that”* (Day Hospice Manager, FG 2)

Across sites, challenges were identified in dealing with the transition and blurring of day-services models with associated changes from social to a more medical led model. Concerns regarding resources to sustain both care models and the implications of this upon the care delivered were raised. A blended model of care was perceived to be advantageous as it enabled patients to receive medical treatment quicker, streamlined with acute and primary care services. It also provided another reason to attend day-service, thereby enhancing attendance rates. Participants perceived the medical approach to have a ‘ripple effect’ upon other services, helping reduce pressure on primary care, reduction of emergency attendances and admission to hospital. However, the lack of evidence to substantiate these claims was viewed by some as a key gap in the knowledge base of the economic effectiveness of day-services, making it difficult to demonstrate value to senior managers and commissioners:

*“I think there needs to be some kind of realisation … [that] … day services are making a difference and if it wasn’t for them it could be x amount of patients in hospital costing you x amount...”* (Hospice Manager FG 6)

*“We’ve either prevented the General Practitioner consultation … or we’ve prevented an out-of-hours admission because things have escalated” (*Hospice Consultant, FG6)

*“ … and it comes back to thinking about costs actually. Because if we can advocate and prevent a lot of these situations from happening you know, we talk about what can offer in terms of the overall costs for the health system”* (Community Service Manager FG 4)

Despite the wide variation in service provision, participants agreed that the broad purpose of day-services is to enhance the quality of life, through interventions to ensure comfort, happiness and standard of health, of people with palliative care needs.

#### Unclear referrals, inconsistent patient population and uncertain value

The referral process is unclear, with little clarity surrounding the process and eligibility criteria applied to day-service across the three sites. Several factors that influenced referral were discussed as well as timing of referral. For example, participants recognised that they were dependent on others such as primary care teams, for the viability of their service, causing anxiety. A recurrent theme revealed that staff external to day-services, such as specialist and generalist practitioners, were the primary sources of information, and of referral to the services. Patients referred were often already known to the palliative care team.

*‘ … one of the challenges of day services is actually trying to keep a full complement of patients coming … people come in and they leave for whatever reason … maybe a few people have died, and our numbers are down again and it’s this constant … it’s almost as if the service isn’t fully utilized … ’* (Health Care Assistant FG3)

‘*I think the reason why there isn’t an influx of referrals is because professionals either don’t know about the service or are not aware or they don’t know how to refer, or they don’t know who to refer … one of the biggest limitations of the service is how you go about, you know, educating people* … ’ (Day Hospice Manager FG 2)

Referrals and their timing were also influenced by gatekeepers’ recognition of palliative care need, knowledge and beliefs about day-services and awareness that patients’ requirements could be addressed by such programs. The participants provided several examples of patients and families who could have benefited from day-services but were unable to access them early enough to avail themselves of the benefits, due to the timing or lack of referrals.

*‘ … there is a big piece of work that needs to be done in and around day services to get the message out there … that we’re here, and this is what we do … health professionals are just not 100% sure of what we do.’* (Day Hospice Manager FG 2)

*“By the time the patient was referred they were already very advanced in their illness and the wife had been doing all of the caring and they hadn’t had any support. If he had been referred to us even three months earlier, he would have had such a different experience of care … and health professionals aren’t particularly good at that...”* (Community Services Lead Nurse, FG 4)

Earlier referrals were recommended as a catalyst to good relationships being developed between hospice staff, patients and their families. Strategies to educate and engage gatekeepers were reported to be in progress although this was a resource-heavy activity across the units. Some staff undertook ‘snowball recruitment’ by contacting senior staff to see if they knew of anyone else who would benefit from the service. Other participants reported that they were trying to develop greater links with community, allied health professionals and acute services via dissemination of flyers and open days. Others were open to reassigning traditional hospital services within day hospice such as blood transfusions, dependent upon funding, which helped to develop links and educate a wider remit of gatekeepers and keep the day-service at the forefront of service delivery. One manager explained:

*“..looking at people who’ve been admitted into hospital and then looking at what those people need … and so what I hope I can do this afternoon is say “Look, we’re here, this is what we do, this is who we work with, if you come across somebody either who’s known to us and has fallen off the wheel or whatever you call it! Or somebody who we don’t know but actually fits our criteria then please consider referring””* (Hospice Manager FG 4)

When asked about the patient profile of units, responses revealed that most attendees were aged between 50 and 60 years, presenting with a cancer diagnosis. Participants attributed the age group attendance due to the stereotypical view of day-service. Participants also reported an increase in referrals of patients with non-malignant conditions such as neurological, heart disease, kidney disease and stroke and attributed this change to Marie Curie’s national charity changing identity from cancer focused, to one encompassing all diseases. However, they recognised that palliative care patients are not a homogenous group and day-service as it currently operates, may not be meeting the needs of people of subgroups such as younger, working adults or cultural ethnic groups. One staff nurse said:

*‘It’s definitely generally older people that come along to day services and I think a lot of that is maybe because of social isolation. I don’t know maybe younger peoples’ needs are being met elsewhere or is it that they could benefit but actually don’t because they just associate it being for older people and that puts people off.’* (Staff Nurse FG 3)

*‘I wonder if, for young people, if it is just more the association of the name ‘day services’, they think of it more somewhere where a granny or an elderly parent would go … I don’t really think there’s a lot of support out there for that sort of age group really.’* (Volunteer Complimentary Therapist FG 3)

*“But I sometimes wonder for younger people whether there’s a different mind-set maybe. Certainly, for me … if I had a major diagnosis I really would not want to come anywhere near here, absolutely not*” (Hospice Manager FG 4)

Participants confirmed that referral criteria were unclear, and this contributed to an inconsistent, variable patient population both within and across the research sites.

### Recognising the strengths and challenges

Focus group discussions highlighted several strengths and challenges facing day-services. At an operational level, care was being delivered by a highly committed multidisciplinary expert team. The diversity of skills and the links within the wider hospice system enabled patients to be fast-tracked to specialist palliative care services when required, providing an environment where staff and patients could access specialist advice, on an ad-hoc basis. Having time to talk, enabled the patient to be at the centre of the care and decision-making process. One participant linked the role of day-service to the ‘key worker’ role in coordinating the care and promoting continuity of care for patients within and across specialist and generalist care, saying:

*‘ … I think the changes that we’ve made to integrate day-services and with our community services … also means that continuity throughout their illness … they’re not being passed from pillar to post.’* (Community Services Lead Nurse, FG 4)

Participants believed day-service improved patients’ quality of life by enhancing opportunities for social interaction, access to peer support, and providing them with a sense of purpose within a safe environment in which to express their true feelings:

*‘ … they get as much from the social interaction with each other and the support of each other as they would from us. It’s massive for them … where they are more relaxed to be able to tell you things … ’* (Occupational Therapist FG 1)

*“… a huge component of what day-service is doing is integrating people with other individuals who are going through similar problems and having supportive staff and volunteers there to manage some conversations and discussion. And at the same time allow carers and those at home who are managing on a daily basis to have some time off … or to be seen independently, and to have their thoughts and needs and worries addressed independently of the patient”* (Community Services Manager, FG 4)

*‘ … he [patient] said ‘I get tea and sympathy at home, and people looking at you with pity. Here, I meet people in the same circumstances as I’m in and who understand what it’s like to have a terminal illness … . There is a very poignant statement by a patient, so the social aspect is just as important.’* (Hospice Manager, FG 2)

Participants reported benefits for family caregivers: respite, tailored therapeutic services, support and signposting to additional services. They said day-services introduces patients and family caregivers to the hospice environment, breaks down stereotypes and acts as a bridge to engage them and facilitate timely transition to hospice care:

*“ … . We sort of help to build that bridge, to fill in the gap for patients. Especially for patients who are at home … they’re not in a hospital, they’re not in a hospice and they don’t always know who to go to for* this *or who to go for* that*”* (Occupational therapist, FG 1)

Several challenges were cited. Day-services were described as invisible to many, leading to low uptake, inadequate funding and lack of evidence of effectiveness. There was a belief that the goals and roles of such programs were poorly understood, and their contribution undervalued. Staff reported pressure to demonstrate value for money, yet value was traditionally measured by a tick box approach i.e. numbers attending, or number of assessments undertaken. Many felt this underplayed the true value of day-services, more accurately reflected in patients subjective experience. One participant stated this could be compromised by mission *creep*, with the service responding to demands from commissioners:

*“Mission creep, is where you set off as an organisation doing one thing, so we do end of life care really well but both with commissioners input and with pressure from funding, you end up “oh yes we can do that and we can do that “and before you know it you are so far away from where you started off”* (Hospice Manager FG 2)

*‘We’re very much within financial constraints, the CCGs are stuck with giving money, we do need to prove our worth, so we do need to be thinking on everything that we’re doing with new services … but it doesn’t help us get money so we need to be thinking outside the box as to how we get the money for day-services.’* (Hospice Consultant FG 6)

*‘I think what’s really interesting is that when you ask the question of what we have and what we do, when you talk about these sorts of things, and you can make them real with stories, it’s pretty evident to other people what can be achieved, but trying to make that understandable for the people who actually fund and commission healthcare is actually very difficult … ’* (Consultant, FG 4)

This was exacerbated by a lack of physical and financial resources for developing and expanding the service. Participants reported limited physical space, limited service delivery and this prevented sensitive conversations with patients and family members. Whilst many participants wanted to expand the service, they realised that resources were limited, that not all needs could be met through day-services.

### An uncertain future

Looking forward, participants agreed that greater awareness and standardisation of day-services would help. Some called for more flexible operating times, including weekends, while others agreed that patients should be targeted earlier in their illness trajectory. One manager suggested day-services should be more flexible and open, as to what services they offer and model of care they adopt.

The need to review the referral process to enable earlier access, appropriate referrals, and consistent reassessment to improve retention was identified. It was recommended that patients should be able to self-refer to widen access for the frail elderly. Participants also wanted to see replacement of the ‘value-for-money’ philosophy, to one of widening access to the wider community, by means of increased funding, creation of more places, and promotion of day-services amongst the public. Greater recognition of the value of patients’ perspectives of day hospice was recommended as a more appropriate measure of service impact:

*‘ … I think we need to get more feedback from the patients to have that evidence to give to commissioners … Because it’s not about what the commissioners think we do, it’s about what the patients need. It’s the needs of those patients … ’* (Hospice Manager FG 2)

## Discussion

Palliative care day services reflect the holistic nature of palliative care, in aiming to improve the quality of life of patients and their family caregivers complementing mainstream palliative care, as previously suggested by Vandedale et al. [25] The challenge is to recognise needs that may be addressed via day-services and then to refer patients immediately and integrate day-services into care plans early, even alongside other models of curative intent or life-prolonging treatment.

The lack of standardisation of day services has resulted in variations in function, delivery, model of care and staffing, confirming previous research [15, 25, 28]. Brereton and colleagues [11] noted a lack of consensus on the model and variable outcome measures makes replication or application to specific patient groups challenging. Participants noted that the origins of day-service stemmed from a social model of care that has evolved to combine both social and medical services. The hybrid model was viewed as advantageous as it enabled patients’ physical, and psychosocial needs to be met in line with the holistic nature of palliative care, so having the potential to reduce demand on other health care services. The lack of evidence about the impact of day services on patient outcomes makes it difficult to quantify such beneficial effects and indeed to understand the specific nature of the contribution of palliative care day services. There is a need for the model of day-service and the implications of this upon other services, to be investigated.

The limited use of day-services reflects that found in previous investigations [15, 19, 23]. Here, staff felt pressure to ensure adequate numbers attend, but this must be balanced with the services available. Utilisation was influenced by attrition of patients from the service and the referral process. Whilst the model has changed to include clinical input, the mode of referral has not [8, 15], such that it is reliant on external gatekeepers and their understanding of palliative care day services provision. Lack of knowledge on the part of these gatekeepers, regarding palliative care day services function and the range of patient needs it can meet, also affects the timing of referrals, a key issue in patients accessing appropriate timely care. In particular late referral has implications for the delivery of services but also on the patient and caregiver experience.

As noted previously [15, 28, 34, 42],-day hospice is perceived as being beneficial by service providers to patient and carers’ quality of life, by reducing isolation, providing peer support in a safe environment and respite for carers. Patients can also access nursing care, medical treatment and control and monitoring of specific symptoms. The delivery of medical care was viewed as being beneficial to the wider hospice system of care, playing a role in breaking down fears and enabling coordination of care. Too often day care was misunderstood as an older person’s service. Participants recognised that while current attendees were predominantly older, with a cancer diagnosis they realised that terminal care was not limited to an age group or type of diagnosis. Gaps in the delivery of day care have been identified in the literature [15], prompting calls for a re-thinking the development of day hospice to ensure it is more inclusive to meet the needs of patients from different population groups, with a wider range of diagnoses and expectations.

### Methodological considerations

This study adds to the evidence base from professionals’ perspectives on palliative care day services. The multidisciplinary characteristics of the focus groups enabled us to draw an insight into the different perspectives of clinical and management staff. The adoption of homogenous focus groups (participants in focus groups worked within one site) enabled teams who were used to working together to discuss their own day unit, stimulating self-reflection and debate [38].

However, participants were drawn from only three sites, operated by a single national organisation. The application of inductive thematic analysis rather than applying the themes a priori would support identification of participants views.

## Conclusions

Several issues have emerged which have important implications for the future provision of day-services. There is a need to view these services as a key component of hospice services that responds to the social and medical needs of patients, delivered by a multi-discipinary professional team. However, the potential for patient benefit is challenged by the lack of standardisation and poor visibility of the service among health care professionals.

## Data Availability

Anonymised transcripts have been stored electronically and encrypted. Requests to access the data should be directed to the corresponding author.

## Abbreviations

CCG: Clinical Commissioning Group
FG: Focus groups
UK: United Kingdom
